# Polyphenols of Edible Macroalgae: Estimation of In Vitro Bio-Accessibility and Cytotoxicity, Quantification by LC-MS/MS and Potential Utilization as an Antimicrobial and Functional Food Ingredient

**DOI:** 10.3390/antiox11050993

**Published:** 2022-05-19

**Authors:** Yogesh Kumar, Ayon Tarafdar, Deepak Kumar, Chakkaravarthi Saravanan, Prarabdh C. Badgujar, Aparna Pharande, Sunil Pareek, Olaniyi Amos Fawole

**Affiliations:** 1 Department of Food Science and Technology, National Institute of Food Technology Entrepreneurship and Management, Kundli, Sonipat 131028, Haryana, India; yogeshk@niftem.ac.in (Y.K.); deepkk@niftem.ac.in (D.K.); 2 Department of Food Engineering, National Institute of Food Technology Entrepreneurship and Management, Kundli, Sonipat 131028, Haryana, India; ayontarafdar@icar.gov.in; 3 Livestock Production and Management Section, ICAR-Indian Veterinary Research Institute, Izatnagar, Bareilly 243122, Uttar Pradesh, India; 4 Department of Basic and Applied Sciences, National Institute of Food Technology Entrepreneurship and Management, Kundli, Sonipat 131028, Haryana, India; chakkaravarthi@niftem.ac.in; 5 Laboratory Services Division, Ashwamedh Engineers & Consultants, Nashik 422009, Maharashtra, India; aparna@ashwamedh.net; 6 Department of Agriculture and Environmental Sciences, National Institute of Food Technology Entrepreneurship and Management, Kundli, Sonipat 131028, Haryana, India; 7 Postharvest Research Laboratory, Department of Botany and Plant Biotechnology, University of Johannesburg, Johannesburg 2006, South Africa

**Keywords:** macroalgae, chlorophyll content, in vitro digestibility, MTT assay, fucoxanthin, phloroglucinol

## Abstract

Macroalgae are a rich source of polyphenols, and their ingestion promotes various health benefits. However, information on factors contributing to health benefits such as antioxidants, antimicrobial properties, bioaccessibility, and cytotoxicity is less explored and often unavailable. Therefore, this study aims to investigate the above-mentioned parameters for the brown and green macroalgae *Sargassum wightii* and *Ulva rigida*, respectively, collected from the southeast coast of India. *S. wightii* exhibited higher antioxidant activity and moderate antimicrobial activity against major food pathogens in an agar well diffusion assay and in the broth microdilution method (MIC_50_ being <0.5 mg/mL for all microorganisms tested). Both macroalgae extracts exhibited significantly high bioaccessibility of polyphenols. To evaluate the safety of the extracts, in vitro cytotoxicity by a 3-(4,5-dimethylthiazol-2-yl)-2,5-diphenyl tetrazolium bromide (MTT) assay was carried out on the primary cells: mouse splenic lymphocytes. An almost complete decline in the cell viability was seen at considerably high concentration (50 mg/mL), expressing the reasonably high safety of the extracts. The extracts of both macroalgae were quantified for polyphenols, wherein fucoxanthin (9.27 ± 2.28 mg/kg DW) and phloroglucinol (17.96 ± 2.80 mg/kg DW) were found to be greater in the *S. wightii* apart from other phenolics, like gallic acid, quercetin, vanillin, and ferulic acid. The results signify the tremendous scope for the value addition of *S. wightii* through extraction and purification of polyphenols for its potential exploitation in functional foods and nutraceuticals or as an antimicrobial ingredient in active or smart packaging.

## 1. Introduction

“Let food be thy medicine and medicine be thy food”, espoused by Hippocrates around 2500 years ago, is now gaining momentum due to increased interest in health-promoting foods. An excellent source of health-promoting bioactive compounds is macroalgae which are rich in polyphenols. Due to the higher stability of macroalgae polyphenols than the terrestrial plants, they are said to be superior in preventing oxidative stress compared with other edible plants [1,2]. The polyphenols present in brown and green macroalgae especially have good antibacterial, antifungal, and antiviral activity [3,4]. Edible macroalgae, being rich in nutrients and antioxidants (polyphenols), are consumed worldwide either in raw or processed form. A few of the edible species of brown and green macroalgae that are cultivated on the Indian coastline are *Padina gymnospora*, *Sargassum tenerrimum*, *Turbinaria ornata*, *S. odontocarpum*, *P. boryana*, and *Ericaria amentacea*, and the brown algae *Ulva lactuca*, *U. compressa*, *U. intestinalis*, *U. clathrate*, *Caulerpa racemose* [5]. *P. gymnospora*, *S. tenerrimum*, and *S. odontocarpum* and green algae *U. lactuca*, *U. compressa*, *C. racemosa* have been studied for their antioxidant activity, proximate and nutritional content, etc. Nevertheless, *S. wightii* (brown) and *U. rigida* (green) macroalgae have not been researched in much detail with respect to the type or kind of polyphenols present or their properties, something which could add value to their exploitation and possibly for their better utilization.

While there are ample reports on the screening, quantification, and properties of polyphenols in the different brown and green macroalgae found along the European coast (especially Ireland) [6,7,8], there are highly limited studies on the macroalgae cultivated or found on the Indian coastline. Rajivgandhi et al. [9], in one such piece of research, evaluated the antimicrobial activity of *S. wightii* extract against food spoilage-causing bacteria (*Pseudomonas aeurogenosa*). However, to the best of our knowledge, *S. wightii* and *U. rigida* have not been tested against prominent food pathogens (e.g., *Salmonella typhimurium*, *Staphyococcus aurues*, and *Escherichia coli*) or against food spoilage microbes like *Bacillus subtilis*. Therefore, one of the aims was to examine the antimicrobial activity of *S. wightii* and *U. rigida* against these microorganisms. Furthermore, cytotoxicity studies are important for checking the safety of macroalgae extracts to know the safe concentrations that can be used for different functional applications. Human blood lymphocytes and mice splenic lymphocytes have been used to study the cytotoxicity of macroalgae extracts [10,11]. *Padina pavonica* (brown algae collected from the Urla coastline of Turkey) was shown to have significantly high cytotoxicity toward human blood lymphocytes [10]. Again, not many studies were found on cytotoxicity evaluation of extracts from the macroalgae *S. wightii* and *U. rigida*.

Digestion and absorption of macroalgae polyphenols are affected by the pH, inorganic salts, and digestive enzymes in the gastrointestinal tract [12,13]. In vitro simulated gastric and intestinal fluids are used for the bioaccessibility analysis of phytochemicals. Huang et al. [12] have reported good bioaccessibility of brown macroalgae (*Sargassum* genus) phenols under simulated intestinal digestion, which was determined in terms of the total phenolic content (TPC). Currently, there are no reports on the bioaccessibility of phenols or polyphenols present in the two selected edible macroalgae—*S. wightii* and *U. rigida* (either on the Indian coastline or from any other coast of the world)—and hence, we have evaluated the same.

The nutraceutical potentials of macroalgae differ with variations in the species, season, temperature, climate, salinity of water, amount of sunlight, harvesting time, and even the part of the macroalgae used (e.g., thallus, blades, or leaves) [13]. Moreover, differentiation in the concentration and the polarity of a solvent also plays a major role in extracting phytochemical compounds. For instance, Rajauria et al. [14] studied the effect of methanol concentration (0–100%) on the extraction of phytochemicals present in *Himanthalia elongata* (Countey Donegal, Ireland) and reported the highest TPC and total flavonoid content (TFC) in 60% methanolic extract. Airanthi et al. [15] also recorded that seven different species of Japanese edible brown macroalgae showed higher phytochemical contents in a polar solvent (absolute methanol) than other organic solvents (e.g., chloroform, ethanol, acetone, *n*-hexane, and diethyl ether). With respect to the selected macroalgae in this study, such data about a suitable solvent and its concentration for the extraction of bioactivities is limited [1,16]. Furthermore, we believe that periodic re-evaluation of polyphenols and their characteristics is a need of the hour considering the ever changing climatic conditions (global warming), water salinity, pollution, etc. The literature about the quantification of polyphenols in these edible macroalgae found on the Indian coast is scarce. Additionally, the crucial functional (antimicrobial, cytotoxicity, and bioaccessibility) and spectral properties of the polyphenols present in these macroalge have not been evaluated. This has led to a lack of popularity and underutilization of the macroalgae among Indian masses on account of a poor scientific background and inadequate publicity of the latent potential of the macroalgae. This also makes their use in the commercial value-added food products very rare.

Considering this, the present study was undertaken to investigate the functional attributes of *S. wightii* and *U. rigida*, including the phytochemical content (TPC and TFC) and antioxidant activity (radical scavenging activity, total antioxidant activity, chlorophyll content and antimicrobial activity against major food pathogens). Furthermore, polyphenols present in these macroalgae responsible for the functional attributes were quantified through LC-MS/MS. Moreover, the in vitro digestibility and in vitro cytotoxicity of the polyphenols in both macroalgae were determined and reported for the first time.

## 2. Materials and Methods

### 2.1. Chemicals

Ascorbic acid, sodium hydroxide, nutrient agar, trypsin soy broth, Folin-Ciocalteu’s phenol reagent, bile extract, and a red blood cell (RBC) lysis buffer were purchased from Hi-Media (Mumbai, India). Pepsin and lipase from porcine, porcine pancreatin, fucoxanthin (≥95%, HPLC), phloroglucinol (≥99%, HPLC), 2,2-diphenyl-1-picrylhydrazyl (DPPH), quercetin, gallic acid, 6-hydroxy-2,5,7,8-tetramethylchroman-2-carboxylic acid (Trolox), acetone, methanol acetonitrile, antibiotic-antimycotic solution, and 3-(4,5-dimethylthiazol-2-yl)-2,5-diphenyltetrazolium bromide (MTT) were procured from Sigma-Aldrich (Bangalore, India). Ferulic acid (≥98%) and vanillin (≥99.4%) were purchased from SRL & Molychem (Mumbai, India), respectively. Dulbecco’s Modified Eagle’s Medium (DMEM) and fetal bovine serum (FBS) via Gibco were procured from Thermo Fischer (New Delhi, India). The methanol (Avantor, Mumbai, India) was LC-MS grade, while all other chemicals used were of an analytical grade.

### 2.2. Procurement and Processing of Macroalgae

Green (*Ulva rigida*) and brown (*Sargassum wightii*) macroalgae were obtained from the macroalgae traders of the Kanyakumari and Mandapam districts of Tamil Nadu, India. The macroalgae were cleaned with tap water and shade-dried up to a moisture content of 21.53 ± 0.05% (wet basis) at 26 ± 2 °C. The shade-dried macroalgae were powdered to a 850-µm size using a mixer-grinder (Sujata, India). The dried macroalgae powder was stored at −20 °C in airtight LDPE bags.

### 2.3. Estimation of the Chlorophyll Content

The chlorophyll content was estimated by the method described by Syad et al. [17]. The expression of the chlorophyll A (C_a_) and chlorophyll B (C_b_) contents was used for calculation as described in Equations (1) and (2):

(1) $$C_{a}=15.65\;\left(A_{666}\right)-7.340\;\left(A_{653}\right)$$

(2) $$C_{b}=27.05\;\left(A_{653}\right)-11.21\;\left(A_{666}\right)$$

The chlorophyll content was expressed as µg/g of fresh weight (fw) of macroalgae.

### 2.4. Antimicrobial Activity

The antimicrobial activity was performed using the agar well diffusion method described by Rajivgandhi et al. [9]. One-day-old inoculated well-grown *Salmonella typhimurium*, *Escherichia coli*, and *Staphylococcus aureus* were streaked on the nutrient agar plates with the help of sterile buds. One hundred µL of the crude extract (100 mg/mL concentration) was used. Ethanol extraction of the macroalgae was carried out for 24 h in a soxhlet apparatus. Briefly, the shade-dried macroalgae were extracted in the ethanol solvent (1:50 *w*/*v*) in a soxhlet apparatus for 24 h at 70 °C. The extracted samples were filtered using Whatmann filter paper No. 1, and the filtered extarct was evaporated using a rotary evaporator (Butchi, Switzerland) at 40 °C. After extraction, each dried extract was then redissolved in the ethanol (100 mg/mL), whereas the absolute methanolic extracts were prepared in an incubator shaker at 37 °C for 24 h, followed by evaporation under a vacuum (Butchi, Switzerland). The extracts were stored at −80 °C.

#### Percentage Inhibition of Macroalgae Extracts

The percentage of inhibition of the ethanolic extract was determined using a broth microdilution method previously described by Boisvert et al. [18]. The food pathogenic bacteria (viz., *S. aureus*, *S. typhimurium*, and *E. coli*) and food spoilage-causing bacteria *B. cereus* were selected, considering the probable futuristic use of the macroalgae extract for antimicrobial food packaging applications. The concentrations used for this assay were 0.6, 0.8, 1, 2, and 4 mg/mL for the ethanolic extract of both macroalgae [6].

### 2.5. Analysis of Phytochemical Content and Antioxidant Activity

Extraction was carried out as per the protocol of Wang et al. [19] with minor modifications. Five grams of liquid nitrogen-dried macroalgae powder (850 µ) was mixed with 100 mL of each solvent (0% methanol or aqueous, 20% methanol, 40% methanol, 60% methanol, 80% methanol, and 100% or absolute methanol). The mixture was incubated in a shaker incubator (New Brunswick Scientific, Eppendorf AG, Germany) at 100 rpm for 24 h at 37 °C. The mixture was then centrifuged at 1274× *g* (3500 rpm) at 4 °C for 10 min and filtered with the help of Whatman No. 1 paper. The filtered extract was evaporated in a rotary evaporator (Butchi, Switzerland) to concentrate the macroalage extract into the corresponding solvents, followed by drying of the extracts in an oven at 37 °C. After extraction, each dried extract was then redissolved in its corresponding solvent (1 mg/mL) and stored at −20 °C.

The phytochemical content (TPC and TFC) and antioxidant activity (DPPH-free radical scavenging activity and ferric-reducing antioxidant power (FRAP)) were determined according to Kumar et al. [20]. The total antioxidant activity (TAA) was evaluated according to the methods of Prieto et al. [21]. Briefly, 0.3 mL of seaweed extract (1 mg/mL concentration) was mixed with the reagent solution (0.6 M sulfuric acid, 28 mM sodium phosphate, and 4 mM ammonium molybdate (1:1:1)) in a microcentrifuge tube. The tubes were capped and incubated in a water bath at 95 °C for 90 min. The samples were cooled to room temperature, and the absorbance of the samples was measured at 695 nm against a blank. A typical blank solution contained 0.3 mL of the same sample solvent and a 3-mL reagent solution. The total water and lipid-soluble antioxidant activities were expressed as mg ascorbic acid equivalent per g of extract (mg AAE g^−1^) and mg BHT equivalent per g of extract (mg BHT g^−1^), respectively.

### 2.6. Bioaccessibility of Macroalgae Phenols and Polyphenols

The bioaccessibility of the polyphenols present in both Indian edible macroalgae was estimated by an in vitro simulated digestion method. The macroalgae methanolic extracts were subjected to consequent simulated gastric fluid (SGF) and intestinal fluid (SIF) treatments to mimic the human gut system. The assay was carried out as previously described by Huang et al. [12] for SGF preparation and gastric phase digestion (1 mL ethanolic extract of macroalgae (as discussed in Section 2.5) was added to 20 mL SGF) as well as SIF preparation and intestinal phase digestion (10 mL of each treated sample with SGF was added to 10 mL SIF). The supernatant obtained at the end was further subjected to estimating the final bioaccessibility.

The bioaccessibility of the macroalgae phenols and polyphenols was determined by estimating the phytochemical content and antioxidant activity (of the gastric and intestinal phase supernatants), which was carried out as described in Section 2.5. The bioaccessibility (%) of the macroalgae phenols was calculated using the equation described by Kumar et al. [22]:

*Bioaccessibility* (%) = (*A_digested_*/*A_nondigested_*) × 100 (3)

where *A* is the TPC and FRAP of the digested and non-digested samples.

### 2.7. Cytotoxicity of Macroalgae Extracts by an MTT Assay

#### 2.7.1. Preparation of Mouse Lymphocytes

Swiss albino female mice (4–6 weeks old) were used for the isolation of splenic lymphocytes. Experiments were performed after obtaining prior permission of the Institutional Animal Ethics Committee (IAEC) (proposal approval no. 6/IAEC/2018) and as per the guidelines of the Committee for the Purpose of Control and Supervision of Experiments on Animals (CPCSEA). The mice were housed in polystyrene cages with ad libitum access to filtered tap water. The room was maintained under a 12/12 h light-dark cycle. The spleen was collected from a mouse having no history of clinical symptoms. Preparation of the mouse lymphocyte single-cell suspension was performed according to the method of Badgujar et al. [23].

#### 2.7.2. Determination of Cytotoxicity

The cytotoxicity assay was performed according to Ahmadi et al. [24] with slight modifications. DMEM was employed as a culture medium in this study, and ethanolic macroalgae extracts (*S. wightii* and *U. rigida*) were used at concentrations of 0.25, 0.5, 1, 5, 10, and 50 mg/mL that were dissolved in DMEM with 5% dimethyl sulfoxide (DMSO). Briefly, 50 µL of lymphocytes (2 × 10^6^ cell/mL) maintained in a suspending medium were transferred to the 96-well plates and were exposed directly to 50 µL of each macroalgae extract in triplicate. Cells without the extract acted as a control. The plate was then transferred to a CO_2_ incubator (5% CO_2_ humidified condition) for 24 h at 37 °C. The supernatant was discarded after centrifugation (600× *g*/10 min at 20 °C), and precipitate washing was performed with 200 µL phosphate buffered saline (PBS). Ten µL of MTT solution (5 mg/mL) was added to the wells and incubated for 4 h. The MTT was removed after centrifugation (600 g/10 min), and the formazan crystals were resuspended with 100 µL DMSO. The absorbance was read at 570 nm (630-nm reference wavelength) using a multimode microplate reader (Spectramax M2e, Molecular Devices, San Jose, CA, USA). Cell viability was calculated as the absorbance of treated cells/absorbance of control cells × 100.

### 2.8. Liquid Chromatography–Mass Spectrometry LC-MS/MS Analysis

The macroalgae samples were extracted as previously described by Agregan et al. [25] with slight modifications. Extraction was carried with the prepared macroalgae powder and acetone:methanol (7:3, *v*/*v*) as solvents in a liquid/solid ratio of 30 g/g on a magnetic stirrer for 5 min. The samples were then subjected to bath ultrasonication (MRC, Israel) for 10 min at room temperature, followed by centrifugation (2000× *g*/10 min). The supernatant obtained was filtered through a 0.22-µm nylon filter. All samples were analyzed in triplicate by LC-MS/MS as per the conditions described by Kumar et al. [26].

### 2.9. Statistical Analyses

The data are expressed as mean ± SD, n = 3. The chlorophyll data were analyzed with an independent sample *t*-test. All data were analyzed by one-way ANOVA followed by Duncan’s multiple comparisons post hoc test and an independent sample *t*-test using SPSS statistical software v.20 at a 5% level of significance (*p* < 0.05). Graphs were drawn with Graph Pad Prism v.5 software.

## 3. Results

### 3.1. Chlorophyll Content

The antioxidant nature of chlorophyll prevents the oxidation of linoleic acid and lipid peroxidation of low-density lipoprotein (LDL), and its estimation is therefore a crucial parameter for macroalgae research [27]. *S. wightii* showed lower quantities of chlorophyll than those of *U. rigida* (Table 1). In previous studies, it was reported that the lower quantities of chlorophyll a may be due to the light and temperature stratification. The carotene pigment level might be different in the same and different species, and as a result, they absorb the excess amount of light, eventually decreasing chlorophyll content [28]. Furthermore, this difference could be due to the extraction of chlorophylls, which has been reported to depend on the polarity of the solvents [29]. Garcia-Perez et al. also reported that some brown macroalgae (*Himanthalia elongata* and *Undaria pinnatifida*) extracted in the acetone solvent had the highest content of chlorophyll a compared with those of the methanol and ethanol solvents [29]. Our results are in line with the findings of Syad et al. [17], who reported a 6.56-µg/g fw chlorophyll a content in *S. wightti* (Gulf of Mannar, India). No research could be found to compare the chlorophyll a and b contents reported in *U. rigida* from the Indian coast or from any other coast in the world for that matter. However, the lower values of chlorophyll a (1.58 µg/g fw) and chlorophyll b (1.89 µg/g fw) in another green macroalga (*Gelidiella acerosa*) sourced from the same coast (i.e., Gulf of Mannar, India) have been reported earlier [17].

### 3.2. Antimicrobial Activity

**Figure 1:**
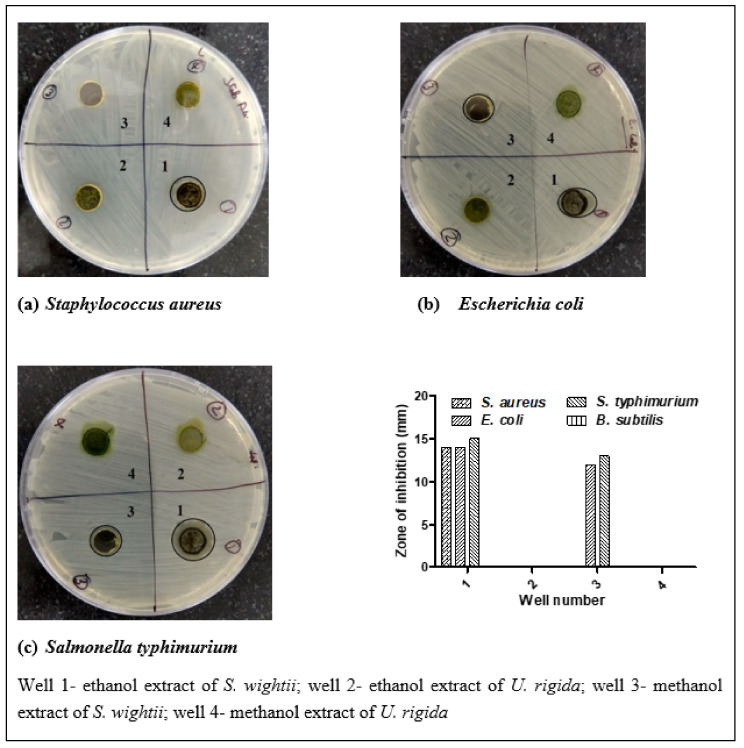
Antimicrobial activity of ethanol and methanol extracts of *S. wightii* (wells 1 and 3) and *U. rigida* (wells 2 and 4), respectively, against *S. aureus* (**a**), *E. coli*, and (**b**) *S. typhimurium* (**c**).

Figure 1 shows the zones of inhibition. The ethanolic extract of *S. wightii* showed strong antimicrobial activity against *S. aureus* (14 mm) and *S. typhimurium* (15 mm), while both the ethanolic and methanolic extracts revealed potent antimicrobial activity against *E. coli* (14 and 12 mm, respectively). Rajivgandhi et al. [9] reported a 12-mm zone of inhibition for the ethanol extract of *S. wightii* against the food spoilage microbe *Pseudomonas aeruginosa*, which is similar to the findings of this study. Moreover, our results are comparable to that of Suganya et al. [30], who reported high antimicrobial activity for *S. wightii* (ethanol and methanol extracts) against *P. aeruginosa* (20 and 13 mm, respectively), *V. parahaemolyticus* (23.06 and 17.35 mm, respectively), and *E.* *coli* (21 and 17 mm, respectively). Another brown macroalgae (*Laminaria japonica*) has been shown to exhibit antimicrobial activity against gram-positive bacteria (*Listeria monocytogenes, Bacillus subtilis*, and *Micrococcus luteus*) [31]. In our study, the antimicrobial activity of *S. wightii* was promising, but that of *U. rigida* was not. These results are almost in agreement with Bansemir et al. [3], who described how the dichloromethane extract of *U. rigida* (Faro, Portugal) has very weak antimicrobial activity against *Vibrio anguillarum* (2.3 mm) and *Pseudomonas anguilliseptica* (0.8 mm).

The broth microdilution assay is robust and more accurate than other standard methods for determining the percentage of inhibition of pathogens or spoilage-causing food microbes. The results of the percentage of inhibition are presented in Table 2. In general, the ethanol extracts of *S. wightii* were found to have a more potent antimicrobial effect, especially at lower concentrations, in almost all of the four strains of bacteria tested than that of *U. rigida.* The highest concentration tested (i.e., 4 mg/mL) showed very strong inhibition against *S. typhimurium* (91.15%), *S. aureus* (88.07%), *E. coli* (87.32%), and *B. subtilis* (85.01%). It was evident that for all the strains of bacteria, the MIC_50_ of *S. wightii* was lower than 0.5 mg/mL (<500 ug/mL) except for *S. typhimurium*, wherein it was >0.65 mg/mL. Our results are in agreement with Boisvert et al. [18], who reported weak antimicrobial activity for *Ascophyllum nodosum* and *Saccharina longicruris* ethanolic extracts (0.25-mg/mL concentration) against *E. coli* with 37.6% and 10.2% inhibition, respectively. Kadam et al. [32] showed antimicrobial activity in the laminarin extract of a brown macroalga (*Laminaria hyperborean)* against *E. coli* (13.1–45.6%), *S. typhimurium* (13.1–33.4%) and *S. aureus* (5.3–66.8%), representing weak to moderate inhibition intensities.

These results strongly corroborated the maximum zones of inhibition obtained with the *S. wightii* ethanolic extracts. Phenolic compounds of macroalgae extracts have been reported to be responsible for antimicrobial activity [6]. The remarkable antimicrobial activity of *S. wightii* holds significance for its effective utilization in active and smart food packaging and for direct incorporation in food products, and it could prove to be a good natural antimicrobial alternative. In the past, researchers have successfully shown that macroalgae extract can be used in packaging films for their antimicrobial and antioxidant role. In one such example, *Ascophyllum nodosum* (brown macroalga) extract (25 mg/mL and 50 mg/mL water extract of *A. nodosum*) was incorporated to prepare gelatin and sodium caseinate films, which had significantly higher antioxidant and antimicrobial activity than the control films [32]. Recently, macroalgae and macroalgae extracts were successfully incorporated into different food products as antimicrobial agents (viz., *Ulva* acidic extract (1000 mg/kg) added in pork patties, *A. nodosum* methanolic extract (0.5%) added in raw cow milk and yoghurt, and *S. sagamianum* extract (0.25–0.75%) incorporated in bread) [33]. Stévant et al. [34] have reported that *Palmaria palmata* reduced or inhibited the microorganism growth during storage for 126 days at a 6% moisture level, which helps in eliminating the fishy aroma of seaweed.

### 3.3. Phytochemical Content and Antioxidant Activity

#### 3.3.1. Phytochemical Analysis

**Figure 2:**
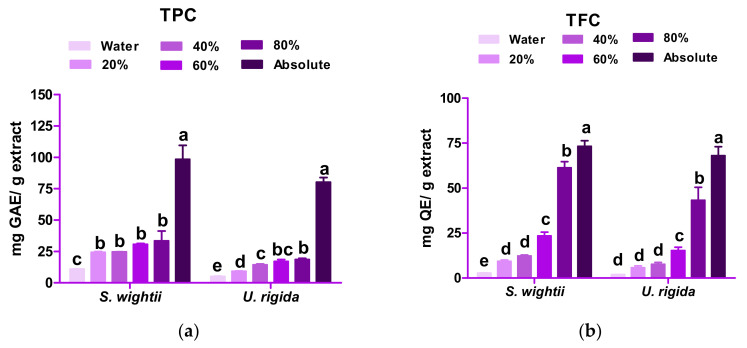
Total phenolic content (TPC) (**a**) and total flavonoid content (TFC) (**b**) of *S. wightii* and *U. rigida* in different concentrations of methanol. Data are expressed as mean ± SD (n = 3). Mean values bearing different superscript letters (a,b…) differ significantly at *p* < 0.05 in a Duncan’s multiple comparison post hoc test. TPC = total phenolic content; TFC = total flavonoid content; GAE = gallic acid equivalent; QE: quercetin equivalent.

The TPC and TFC of *S. wightii* and *U. rigida* varied significantly (*p* < 0.05) with different concentrations of methanol (Figure 2). With an increase in the methanol concentration (20% to absolute), significant increases in the TPC and TFC of both macroalgae were observed. The extracts prepared in the absolute methanol showed significantly higher (*p <* 0.05) TPC and TFC values than the aqueous extracts. *U. rigida* showed a significant difference in TPC among the 20%, 40%, and 80% methanolic extracts. Similar results were reported by Airanthi et al. [15] in five brown macroalgaes in the Hakkaido prefecture of Japan (*Sargassum horneri* and other genera). According to Dang et al. [35], the TPCs of two brown macroalgae (*S. linearifolium* and *S. podocanthum*) were lower and that of *S. vestitum* was higher than that of *S. wightii* in 70% ethanolic extract. Kumar et al. [20] suggested that in *S. wightii*, a polyphenol (phlorotannin) in a methanol extract underwent structural changes upon polymerization, due to which different subunits (fucols, fucophlorethols, eckol, and fuhalols) were formed, increasing the phenolic content. The TFC in the absolute methanolic extract (73.21 mg quercetin equivalent (QE)/g extract) of *S. wightii* was found to be significantly higher (*p* < 0.05) than the aqueous extract (2.75 mg/g extract). Similarly, in *U. rigida*, the TFC was 68.03 mg QE/g in the absolute methanol, while it was 1.90 mg QE/g in the aqueous extract. Rajauria et al. [14] also reported a higher TFC (109.8 ± 2.68 mg QE/g) in the 60% methanolic extract than that of the absolute methanol for the Irish brown macroalga (*Himanthalia elongata*), which is contrary to the TFC of *S. wightii* in the present study.

#### 3.3.2. Antioxidant Activity

**Figure 3:**
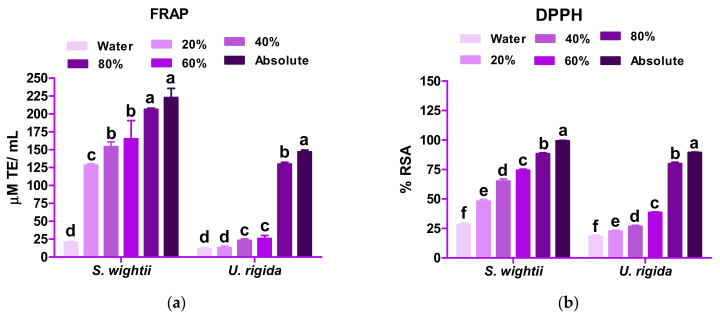

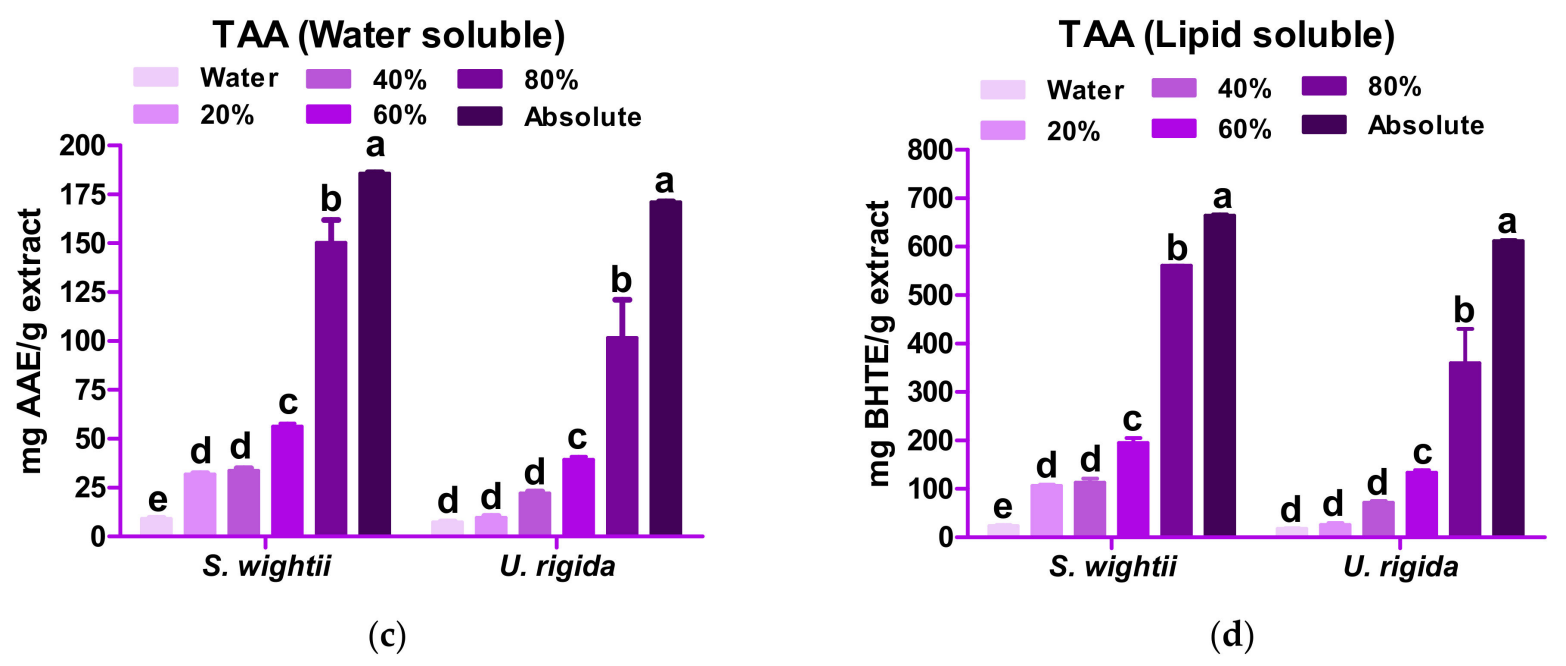
Ferric-reducing antioxidant power (FRAP) (**a**), percentage of radical scavenging activity (DPPH) (**b**), and total antioxidant activity (water soluble (**c**) and lipid soluble (**d**)) of *S. wightii* and *U. rigida* in different concentrations of methanol. Data are expressed as mean ± SD (n = 3). Mean values bearing different superscript letters (a,b…) differ significantly at *p* < 0.05 in a Duncan’s multiple comparison post hoc test. FRAP = ferric-reducing antioxidant power; DPPH = 2,2-diphenyl-1-picrylhydrazyl; TAA = total antioxidant activity; TE = Trolox equivalent; RSA = radical scavenging activity; AAE = ascorbic acid equivalent; BHTE = butylated hydroxy toluene equivalent.

Figure 3 depicts the results of FRAP, DPPH, and water-soluble and lipid-soluble TAA. For both macroalga, their antioxidant activity was found to be directly proportional to the concentration of methanol, indicating that high polarity molecules are responsible for radical scavenging.

The absolute methanolic extract of *S. wightii* showed a significantly higher (*p* < 0.05) FRAP (222.89 ± 12.83 µM Trolox Equivalent (TE)/mL) than that of the aqueous extract (20.67 ± 1.12 µM TE/mL). In the case of *U. rigida*, the FRAP value of the absolute methanolic extract (147.06 ± 2.48 µM TE/mL) was found to be significantly higher (*p* < 0.05) than that of the aqueous extract (11.50 ± 1.52 µM TE/mL). Matanjun et al. [36] also reported a higher FRAP value (methanolic extract) for *Sargassum polycystum* (brown macroalga) than that of the green macroalga *Caulerpa racemose*, which is comparable to the present study. Methanol could form a complex with the phloroglucinol (polyphenol) present in the macroalga, resulting in higher antioxidant activity [14].

*S. wightii* and *U. rigida* showed significantly higher (*p* < 0.05) DPPH radical scavenging activity (99.28 ± 0.36 and 89.38 ± 0.40% RSA, respectively) in the absolute methanolic extracts. The results of this study coincide with the findings of Do et al. [37]. In the brown macroalga, fucoxanthin, sterols, polysaccharides (fucoidan), protein, and sugar compounds majorly contribute to the RSA [20], whereas the green macroalga contain chlorophyll, which contributes to its rich antioxidant activity [38].

Among the two macroalgae studied, *S. wightii* showed a higher TAA (both for water-soluble and lipid-soluble activity) than *U. rigida* in both the extracts tested (Figure 3d). Yildiz et al. [16] reported that *U. rigida* from the Marmara sea coast of Turkey showed a total water-soluble activity of 375.59 µmol AAE/g fw and total lipid-soluble antioxidant activity of 130.91 µmol α-tocopherol/g fw, which was significantly lower than the corresponding antioxidant values of *U. rigida* observed in this investigation. The total antioxidant activity is said to be related to the substantial quantities of polyphenols present in the macroalgae [16]. The results obtained in our study correlate well with the statement made by Yildiz et al. [16], which itself has been shown to be dependent on the solvent and sample matrix.

### 3.4. Bioaccessibility of Macroalgae Polyphenols

The bioaccessibility of the macroalgae polyphenols after in vitro digestion is reported in terms of the TPC and FRAP. The results are presented in Table 3. It can be seen that the bioaccessibility of the *S. wightii* polyphenols was significantly higher (*p* < 0.05) after simulated in vitro gastrointestinal digestion (TPC: 39.91 ± 4.01% and FRAP: 25.64 ± 1.73%) than that of *U. rigida*.

There is very scarce information available regarding the bioaccessibility of macroalgae phenols, and the majority of the research about the bioaccessibility of macroalgae covers heavy metals. Only two related reports were found on the bioaccessibility of macroalgae phenols. Francisco et al. [39] reported on the bioaccessibility of freeze-dried *Fucus spiralis* (a brown macroalga) with 22.4% TPC and 59.5% FRAP. On the other hand, in a study on different types of macroalga species, the bioaccessibility values were higher after SID (15–42.5 mg GAE/g of TPC) than that of SGD (5–25 mg GAE/g of TPC) [12]. These findings are in parallel to the results obtained in the present study for *S. wightii*. On a slightly different note, Pimentel et al. [40] reported the bioaccessibility (28.34–32.81 mg TE/g of FRAP) of hydrolysates generated from the protein isolates extracted from a red macroalga (*Porphyra dioica*) after SGID. The probable reason for the higher values seen after intestinal digestion in this study may be attributed to the exposure of phenolic compounds to the gastric and intestinal pH environment where ester bonds are hydrolyzed, releasing gallic acid compounds, which have a high antioxidant capacity [41]. Additionally, dietary phenols act as a substrate for various enzymes secreted by gut microorganisms and biotransformed by different metabolic pathways such as hydrolysis, demethylation, reduction, decarboxylation, and dehydroxylation [42], making them more bioaccessible. The in vivo digestibility and bioavailability of these macroalgae phenols in a rat model, as described earlier by Kumar et al. [43], could help to truly substantiate the results for bioaccessibility.

### 3.5. Cytotoxicity Assay

**Figure 4:**
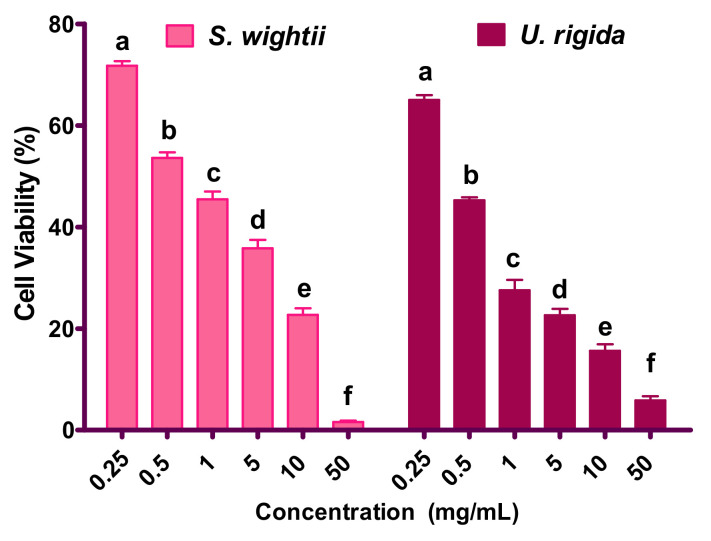
Cytotoxicity of ethanolic extract of *S. wightii* and *U. rigida* at different concentrations (from 0.25 g/mL to 50 mg/mL) measured by MTT assay. Data are expressed as mean ± SD (n = 3). Mean values bearing different superscript letters (a,b…) differ significantly at *p* < 0.05 in Duncan’s multiple comparison post hoc test.

The cytotoxicity results are shown in Figure 4. At a 0.25-mg/mL concentration of the *S. wightii* and *U. rigida* extracts, >70% of the cells were viable, while the highest concentration tested (50 mg/mL) for both seaweeds showed significantly decreased (*p* < 0.05) splenic lymphocyte viability to almost 2% and 5%, respectively. The TC_50_ (concentration that was toxic to 50% of the cells) of *S. wightii* and *U. rigida* against lymphocytes were found to be 0.95 and 0.6 mg/mL. These results indicate that at the higher concentrations of the macroalgae extracts, significant cytotoxicity was produced in the mouse splenic lymphocytes. However, these results need more detailed investigation with respect to whether or not similar results are produced in other cell lines or cells. Nonetheless, the *S. wightii* ethanolic extract was found to be safer at low concentrations up to 1 mg/mL, as more than 50% of the splenic lymphocytes were viable than those of *U. rigida*. Interestingly, the MIC_50_ concentration for the ethanolic extract of *S. wightii* that inhibited the majority of the food pathogens tested was <0.6 mg/mL, giving enough confidence to use the same amount safely for edible and active food packaging applications.

In line with our results, a water extract of the brown macroalga *Padina pavonica* collected from the Urla coastline (Turkey) was shown to be cytotoxic (at 1 mg/mL) to human blood lymphocytes [10]. The *Sargassum* genus extracts have been shown to possess cytotoxicity against various cancer cell lines such as HeLa, MCF-7, HT-29, and PC-3 [44,45]. Our results are also congruent with those of Ahmadi et al., who reported similar cytotoxicity toward human blood lymphocytes with the ethanolic extract of a medicinal plant, *Ziziphora Clinopodioides Lam* [24]. The results of our study are also in agreement with those of Rodeiro et al. [45]. An aqueous-ethanolic extract of the marine angiosperm (sea grass) *Thalassia testudinum* was evaluated for cytotoxicity using primary rat hepatocytes and primary human lymphocytes, wherein a >1-mg/mL concentration was reported to inhibit 50% of cell viability [45]. Further research is required to know the cytotoxicity mechanism of the selected macroalgae extracts.

### 3.6. LC-MS/MS Analysis

**Figure 5:**
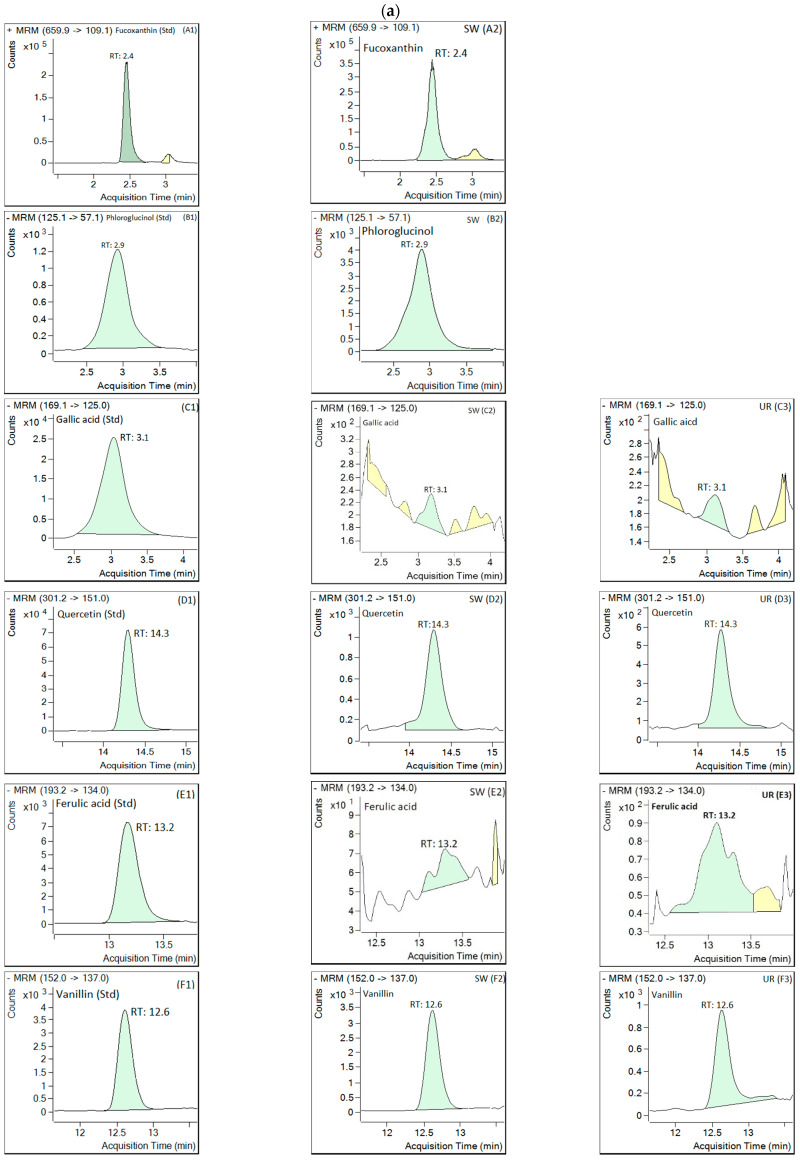

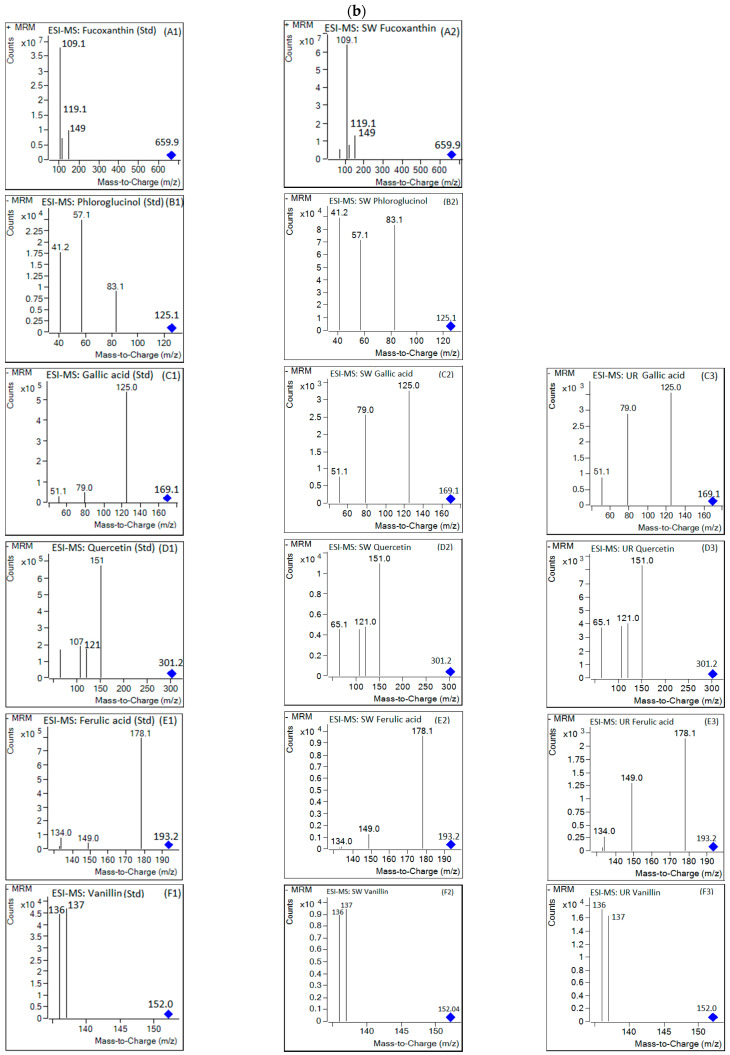

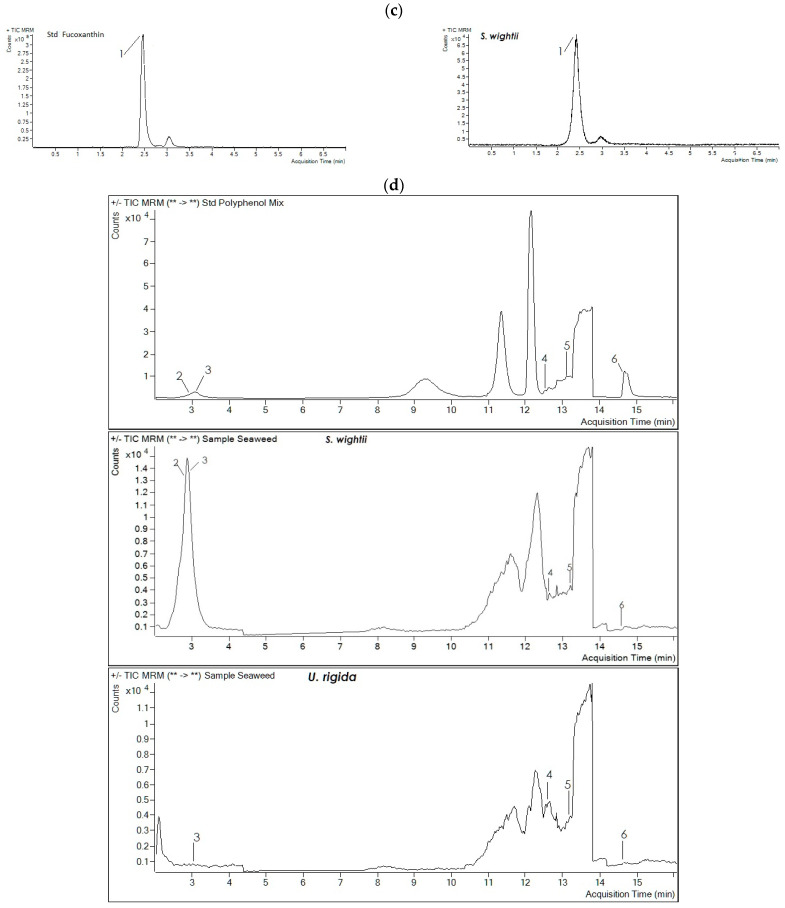
(**a**) LC-MS/MS total ion chromatogram (TIC) spectrum of standard and extracted compounds with retention time of peak. (**b**) ESI-MS spectrum of standard and extracted compounds with product and precursor ions. (A1) Fucoxanthin standard. (B1) Phloroglucinol standard. (C1) Gallic acid standard. (D1) Quercetin standard. (E1) Ferulic acid standard. (F1) Vanillin standard. Extracted compounds are (A2) fucoxanthin, (B2) phloroglucinol, (C2,C3) gallic acid, (D2,D3) quercetin, (E2,E3) ferulic acid, and (F2,F3) vanillin. SW = *Sargassum wightii*, UR = *Ulva* rigida; LC-MS/MS = liquid chromatography with tandem mass spectrometry; TIC = total ion chromatogram; ESI-MS = electron spray ionization–mass spectrometry. (**c**) LC-MS/MS total ion chromatogram (TIC) spectrum of standard (fucoxanthin) and extracted compounds from *S. wightii*. (**d**) LC-MS/MS total ion chromatogram (TIC) spectrum of standard and extracted compunds from *S. wightii* and *U. rigida*. * Acronyms in Figure 5c,d: 1 = fucoxanthin; 2 = phloroglucinol; 3 = gallic acid; 4 = vanillin; 5 = ferulic acid; 6 = quercetin.

The retention time (RT), fragmentor voltage, limit of detection (LOD), limit of quantification (LOQ), collision energy, coefficient of determination (r^2^), and product and precursor ions for the standard compounds were as mentioned in our previous study [26]. The retention time of the peak and ESI-MS spectrum are portrayed in Figure 5, wherein the TIC chromatogram of the standard compounds was comparable to that of the extracted bioactive compounds. Table 4 represents the amount of all bioactive compounds found in both macroalgae.

Figure 5a,b and A1,A2 show a retention time of 2.4 min with a base peak at *m*/*z* 659.9 [M+H]^+^ in the TIC chromatogram, and the product ions were at *m*/*z* 109.1 [M-H-549.8] in the ESI-MS fragmentation of the standard fucoxanthin and the extracted compound of *S.* *wightii*, respectively. These results are in agreement with the reports of Rajauria [46] and Kumar et al. [26] for fucoxanthin ESI-MS fragmentation. The concentration of 9.27 ± 2.28 mg/kg dry weight (dw) of fucoxanthin was confirmed based on ESI-MS fragmentation. Raguraman et al. [47] showed 750 µg/g dw of fucoxanthin in *Padina tetrastromatica* (Indian brown macroalga) using HPLC/MS. In addition, Nunes et al. [48] reported the fucoxanthin contents in 12 brown macroalgae collected from various locations, ranging from 12.2 to 852 µg/g dw. Rajauria et al. [49] reported finding 18.6 mg/g of fucoxanthin in *H. elongate* (Irish brown macroalga) using HPLC/ESI-MS. Jaswir et al. [50] quantified 0.71 mg/g dw fucoxanthin from *S. plagyophyllum* (Malaysian brown macroalga) using RP-HPLC. This study found comparatively lower fucoxanthin amounts in the *S*. *wightii* than those of other brown macroalgae mentioned above. Various environmental factors may be responsible for the difference in the concentrations of bioactive compounds among brown macroalgae, including but not limited to the light intensity, water, season, and salinity [1]. Moreover, higher leaching of polyphenols during extraction depends on the particle size of the dried macroalgae powder, as a smaller particle size leads to higher leaching. Nunes et al. [48] used 74 µm (200 mesh) of macroalgae powder before subjecting it to extraction, while our particle size for the macroalgae was 850 µm (20 mesh).

The MS/MS fragmentation pattern (precursor [M − H]^−^ ions at *m*/*z* 125.1) and peak time of phloroglucinol (2.89 min) were found to be comparable with the standard (2.9 min) (Figure 5a,b and B1,B2). A good quantity (17.96 ± 2.80 mg/kg dw) of phloroglucinol was found in the *S. wightii* and was present in the highest quantity among all the bioactive compounds evaluated. Rajauria [46] also quantified the phloroglucinol (394.1 mg/kg) in *H. elongata* using HPLC/ESI-MS. Phlorotannin in its polymer form, phloroglucinol containing eight interconnected rings, and various derivatives in trimer-to-octamer (fucophloroethol, bifuhalol, phloroethols, and tetrafucol) form in the *Durvillaea antartica* and trimer-to-tetramer form in the *Lessonia spicata* (Chilean brown macroalgae) have been identified using HPLC/MS-MS [51,52]. Mantanjun et al. [36] reported that the special class of phlorotannins such as phloroglucinol might be responsible for higher phytochemical contents in brown macroalgae, leading to greater antioxidant activity than that of green macroalgae. The absence of fucoxanthin and phloroglucinol compounds in the green macroalgae could also be one of the reasons for the low antioxidant activity shown in the spectrophotometric analysis in the present study.

Phenolic compounds such as vanillin, gallic acid, ferulic acid, and quercetin were identified in the extracts of the two tested macroalgae based on MS/MS fragmentation patterns and peak times that were analogous to their respective standards (Figure 5a,b). The extracts of *S. wightii* and *U. rigida* showed gallic acid peaks at 2.8 and 3.1 min, respectively, in the TIC chromatogram with the MS/MS precursor ion (*m*/*z* 169.1) and fragmentation that was comparable with standard gallic acid (3.0 min) (Figure 5a,b (C1,C2,C3)). The fragmentation of the precursor ion (*m*/*z* 169.1) into the product ions *m*/*z* 125 [M-H-44]^−^ and 79 [M-H-90]^−^ was similar to that in the previous report by Abd Ghafar et al. [51]. Similar fragmentation of gallic acid (96.3 µg/g) has also been reported by Rajauria [46] in *H. elongata* using HPLC/ESI-MS. In our investigation, the amount of gallic acid quantified was 0.07 ± 0.00 mg/kg dw in both macroalgae.

In the case of quercetin, the peaks of *S. wightii* and *U. rigida* were detected at 14.28 min and 14.26 min, respectively, in the TIC chromatogram and were comparable to standard quercetin (14.30 min) with MS/MS fragmentation of the precursor ion (*m*/*z* 301.2) (Figure 5a,b (D1,D2,D3)). Fragmentation of the precursor ion (*m*/*z* 301.2) into the product ions (i.e., *m*/*z* 151 [M-H-150]^−^ and *m*/*z* 107 [M-H-194]^−^) was found to be similar to that in the previous report by Pawlowska et al. [53]. Rajauria [46,49] also quantified the quercetin (4.2 mg/kg) in *H. elongata* using HPLC/ESI-MS.

Both macroalgae extracts presented a peak for ferulic acid at 13.34 (*S. wightii*) and 13.24 min (*U. rigida*) that was comparable to standard ferulic acid (13.17 min) in the TIC chromatogram with the same MS/MS fragmentation of the precursor ion (*m*/*z* 193.2) and product ions (i.e., *m*/*z* 149 [M-H-44]^−^ and *m*/*z* 134 [M-H-59]^−^) (Figure 5a,b (E1,E2,E3)). Rajauria [46] reported finding 17.6 µg/g of ferulic acid in *H. elongata*. In contrast, the quantities of ferulic acid determined for *S. wightii* and *U. rigida* were 1.05 ± 0.00 and 1.07 ± 0.00 mg/kg dw, respectively.

The vanillin peaks were identified at 12.62 min for both macroalgae extracts in the TIC chromatogram and were found to be similar to the standard vanillin (12.60 min) with similar MS/MS fragmentation of the precursor ion (*m*/*z* 152) and product ions (i.e., *m*/*z* 137 [M-H-15]^−^ and *m*/*z* 136 [M-H-16]^−^). Similar fragmentation of the precursor and product ions was reported by Khallouki et al. [54]. The *S. wightii* and *U. rigida* contained 1.55 ± 0.31 and 1.23 ± 0.06 mg/kg of vanillin (dw), respectively.

The results obtained through LC-MS/MS analysis corroborate the findings of the phytochemical contents (TPC and TFC) and all antioxidant assays (FRAP, DPPH, and TAA) performed with the absolute methanolic extracts for both of the macroalgae. This study manifests that the phytochemical content and antioxidant activity wre significantly higher in brown macroalgae (methanolic extract), which is presumed to be due to a special class of polyphenols (viz., phlorotannin (phloroglucinol)) and a xanthophyll (fucoxanthin) that are not present in the green macroalgae.

Fucoxanthin is said to contribute more to antioxidant activity than other carotenoids due to the presence of conjugated double bonds with epoxide and acetyl substituent groups adhering to a polyene [49]. Along with these, gallic acid, quercetin, ferulic acid, and vanillin could also help enhance the antioxidant activity of *S. wightii*. Our data have revealed that considerable zones of inhibitions were observed only for *S. wightii*. We believe that bioactive compounds that could be responsible for the distinctive antimicrobial activity of *S. wightii* were the special phenols, namely phloroglucinol and xanthophyll pigments like fucoxanthin.

## 4. Conclusions

This study comprehensively identified the cytotoxicity and antimicrobial activity and quantified the important polyphenols present in two edible macroalgae (*S. wightii* and *U. rigida*) of the southeast Indian coastline. Moreover, the fate of polyphenols (bioaccessibility) post in vitro digestion has been reported for the first time for the selected macroalgae species. *S. wightii* was found to be compositionally richer in polyphenols compared with *U. rigida*. *S. wightii*’s unique phenols (viz., xanthophyll (fucoxanthin) and phlorotannin (phloroglucinol)) could have been responsible for the higher phytochemical content, antioxidant activity, and potent antimicrobial activity. Our study is the first report to show significant antimicrobial activity in the macroalga *S. wightii* against dangerous and high-risk food pathogens (viz., *S. typhimurium*, *E. coli*, *B. subtilis*, and *S. aureus*). Bioaccessibility was found to be higher for *S. wightii* than *U. rigida*. Both macroalgae polyphenols were available for absorption in the gastrointestinal system. Acceptability of nutrient-rich seaweeds is not only scarce among the Indian population in general (compared with the coastal region), but the processed food industry has also not utilized them effectively to develop seaweed-based functional food products. Additionally, agricultural conditions are becoming hostile due to rapid urbanization and climate change, which results in the reduction of agricultural products, and due to the demand for high-nutrient food by consumers, there is a need to develop nutrient-rich food. The results of the present study shall pave the way for the effective utilization of these macroalgae to prepare various value-added products and functional foods. In addition, the polyphenols (fucoxanthin, phloroglucinol, gallic acid, quercetin, vanillin, and ferulic acid) identified and quantified from the macroalgae (*S. wightii* and *U. rigida*) may provide various preventive and therapeutic applications such as anticancer, anti-inflammatory, and antithrombin properties, which can be tested in detail in future works.

## Figures and Tables

**Table 1:** Chlorophyll contents of macroalgae *S. wightii* and *U. rigida*.

Macroalgae	Chlorophyll a (µg/g fw)	Chlorophyll b (µg/g fw)
*S. wightii*	6.41 ± 0.02	ND
*U. rigida*	12.22 ± 0.28 *	13.50 ± 0.14 *

Data are expressed as mean ± SD (*n* = 3). Values bearing * are significantly different (<0.05) from the corresponding row or macroalga tested in an independent sample *t*-test. ND = not detected; fw = fresh weight.

**Table 2:** Percentage inhibition of *S. wightii* and *U. rigida* extracts against selected food pathogens ^1^ and food spoilage bacteria ^2^.

Percent Inhibition								
	*S. aureus* ^1^		*E. coli* ^1^		*S. typhimurium* ^1^		*B. subtilis* ^2^	
Concentration (mg/mL)	SW	UR	SW	UR	SW	UR	SW	UR
0.6	62.96 ± 0.37 ^e^	30.02 ± 0.32 ^i^	67.36 ± 0.23 ^d^	31.99 ± 0.32 ^h^	45.06 ± 0.03 ^g^	30.73 ± 0.14 ^j^	67.39 ±1.83 ^d^	36.03 ± 0.22 ^h^
0.8	65.60 ± 0.23 ^d^	36.44 ± 0.21 ^h^	66.79 ± 0.13 ^e^	31.51 ± 0.16 ^i^	59.80 ± 0.01 ^d^	38.94 ± 1.56 ^h^	69.43 ± 0.37 ^cd^	40.46 ± 3.50 ^g^
1	71.19 ± 0.14 ^c^	37.29 ± 0.21 ^g^	68.51 ± 0.07 ^c^	36.05 ± 0.13 ^g^	64.06 ± 0.01 ^c^	33.56 ± 0.08 ^i^	72.02 ± 0.29 ^c^	44.57 ± 0.29 ^f^
2	74.97 ± 0.18 ^b^	37.42 ± 0.21 ^g^	74.39 ± 0.17 ^b^	46.65 ± 0.17 ^f^	79.81 ± 0.18 ^b^	49.94 ± 0.22 ^f^	76.73 ± 0.21 ^b^	47.45 ± 2.11 ^f^
4	88.07 ± 0.18 ^a^	48.01 ± 0.36 ^f^	87.32 ± 0.24 ^a^	66.51 ± 0.19 ^e^	91.15 ± 0.18 ^a^	51.30 ± 1.35 ^e^	85.01 ± 0.37 ^a^	55.97 ± 3.39 ^e^

SW = *Sargassum wightii*; UR = *Ulva rigida*, compared statistically per microorganism at different concentrations. Data are expressed as mean ± SD, n = 3. Means within columns (for each microorganism) with different letters (^a,b^…) differ significantly (*p* < 0.05) in Duncan’s multiple comparison post hoc test.

**Table 3:** Bioaccessibility (%) of macroalgae polyphenols.

*S. wightii*		*U. rigida*		
Bioaccessibility (%)	SGD	SGID	SGD	SGID
TPC	10.59 ± 0.98 ^c^	39.91 ± 4.01 ^a^	6.88 ± 0.94 ^c^	26.17 ± 1.10 ^b^
FRAP	12.43 ± 0.74 ^c^	25.64 ± 1.73 ^a^	10.70 ± 0.18 ^c^	20.92 ± 0.69 ^b^

Values are presented as mean ± SD. Bioaccessibility percentage values in each macroalga bearing different superscript letters (^a^,^b^…) differ significantly at *p* < 0.05 between columns within each row in Duncan’s multiple comparison post hoc test. SGD = simulated gastric digestion; SGID = simulated gastrointestinal digestion.

**Table 4:** Quantification of bioactive compounds of edible macroalgaes *S. wightii* and *U. rigida* using LC-MS/MS.

Bioactive Compounds	*S. wightii* (mg/kg Dry Weight)	*U. rigida* (mg/kg Dry Weight)
Fucoxanthin	9.27 ± 2.28	ND
Phloroglucinol	17.96 ± 2.80	ND
Gallic acid	0.07 ± 0.00	0.07 ± 0.00
Quercetin	0.04 ± 0.02	0.04 ± 0.00
Ferulic acid	1.05 ± 0.00	1.07 ± 0.00
Vanillin	1.55 ± 0.31	1.23 ± 0.06

ND = not detected. Average concentration of three LC-MS/MS determinations ± SD.

## Data Availability

Data will be provided on demand.
